# Supplementary material for: Concussive Injury in Human Forebrain Organoids Alters Neurogenesis and Neuroimmune Signaling
Source: bioRxiv. 2026 Oct 30:2026.10.29.762930. Preprint. [Version 1] doi: 10.64898/2026.10.29.762930 (PMC13734062; doi:10.64898/2026.10.29.762930)
Supplement: Supplementary file 1 [file media-1.pdf]

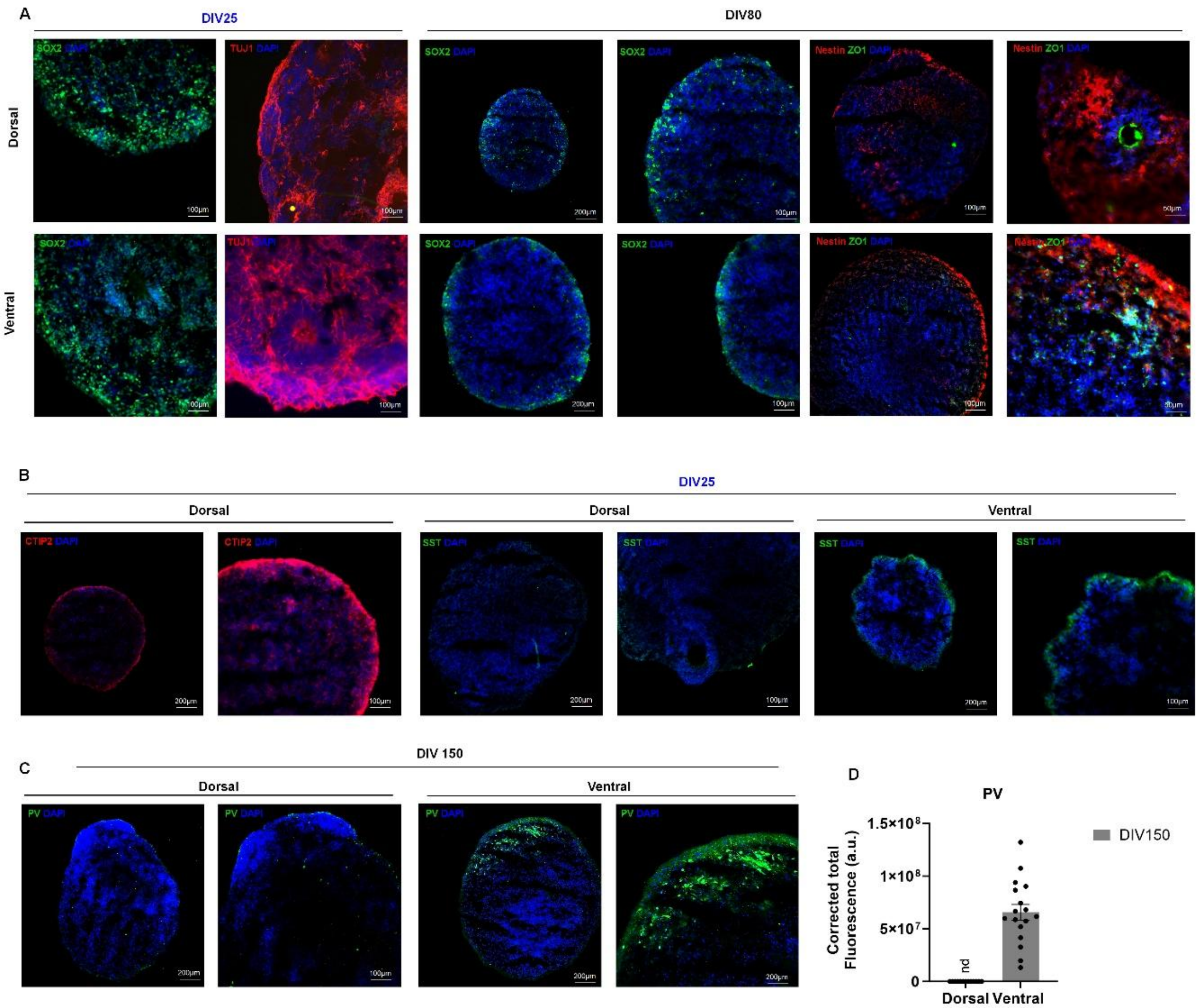

Supplementary Figure 1. Temporal and regional characterization of marker expressions in hFBOs. **(A)** Representative images show SOX2 and TUJ1 expression in dorsal and ventral hFBOs at 25 and SOX2 expression at 80 DIV. Representative images show Nestin and ZO-1 expression in sections from dorsal and ventral hFBOs at 80 DIV, but expression was reduced compared with early stages (see Fig. 1 for expression at 25 DIV). **(B)** Representative images of CTIP2 and SST expression at 25 DIV. SST expression is shown in dorsal and ventral hFBO. CTIP2 was not detected in ventral hFBOs. (**C)** Representative images show expression of parvalbumin (PV) in dorsal and ventral hFBOs at 150 DIV. **(D)** Summary plot comparing PV fluorescence intensity between dorsal and ventral organoids at 150 DIV.

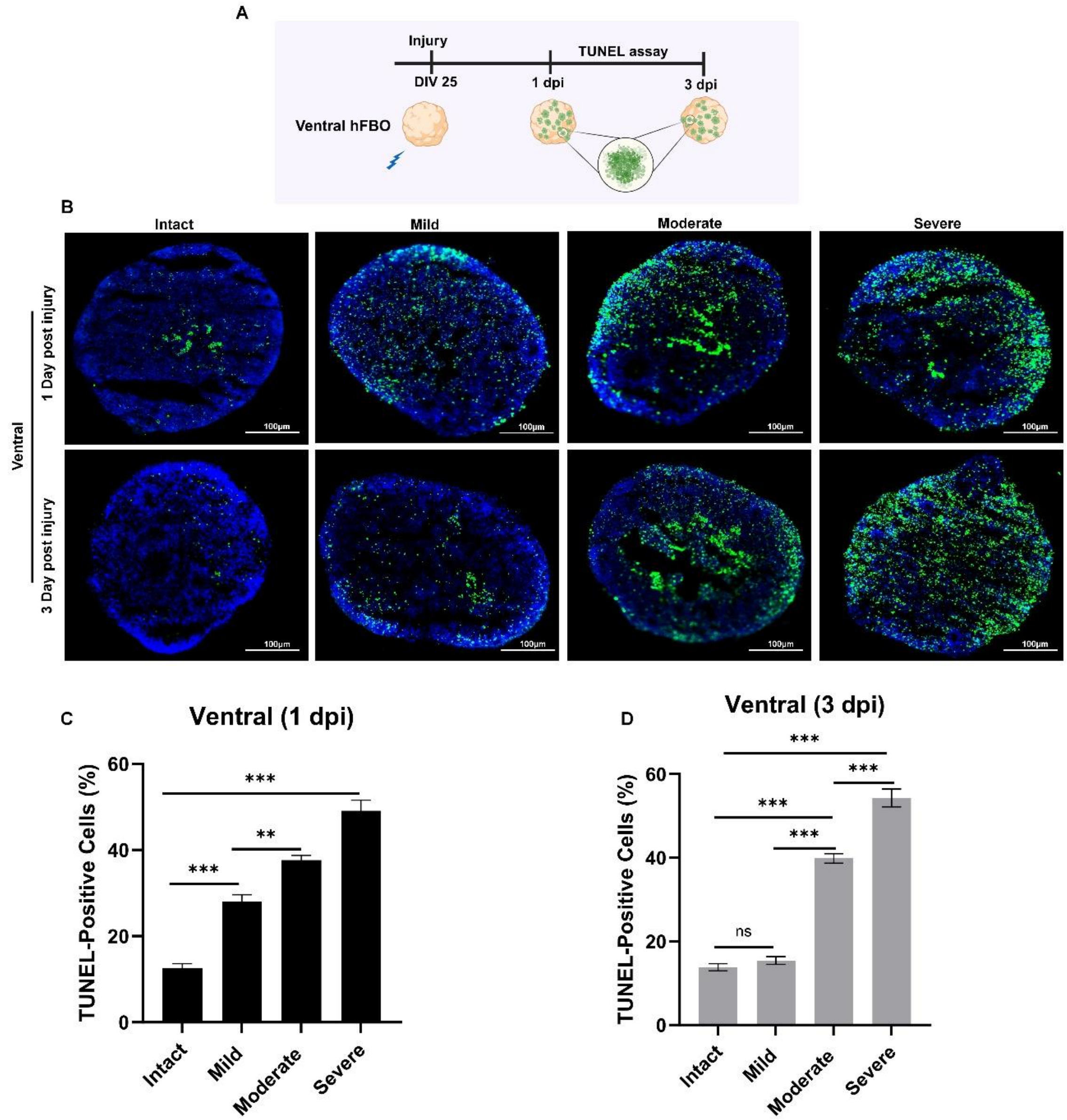

Supplementary Figure 2. Injury severity-dependent cell death in ventral human forebrain organoids. **(A)** Schematic of the experimental paradigm. Ventral hFBOs were subjected to graded concussive injury at 25 DIV, and cell death was assessed at 1 and 3 days post-injury (dpi). **(B)** Representative TUNEL staining of intact and injured ventral hFBOs exposed to mild, moderate or severe injury at 1 and 3 dpi. TUNEL-positive cells are shown in green and nuclei (DAPI) in blue. **(C, D)** Quantification of TUNEL-positive cells at 1 dpi **(C)** and 3 dpi **(D)** demonstrates an injury severity-dependent increase in cell death. n = 8 bins from 5 hFBOs per group, derived from 3 independent batches of 1 hiPSC line. Data are presented as mean ±

SEM and were analyzed by one-way ANOVA followed by Tukey's multiple-comparisons post hoc test. ns, not significant; **p < 0.01; ***p < 0.001; ****p < 0.0001.

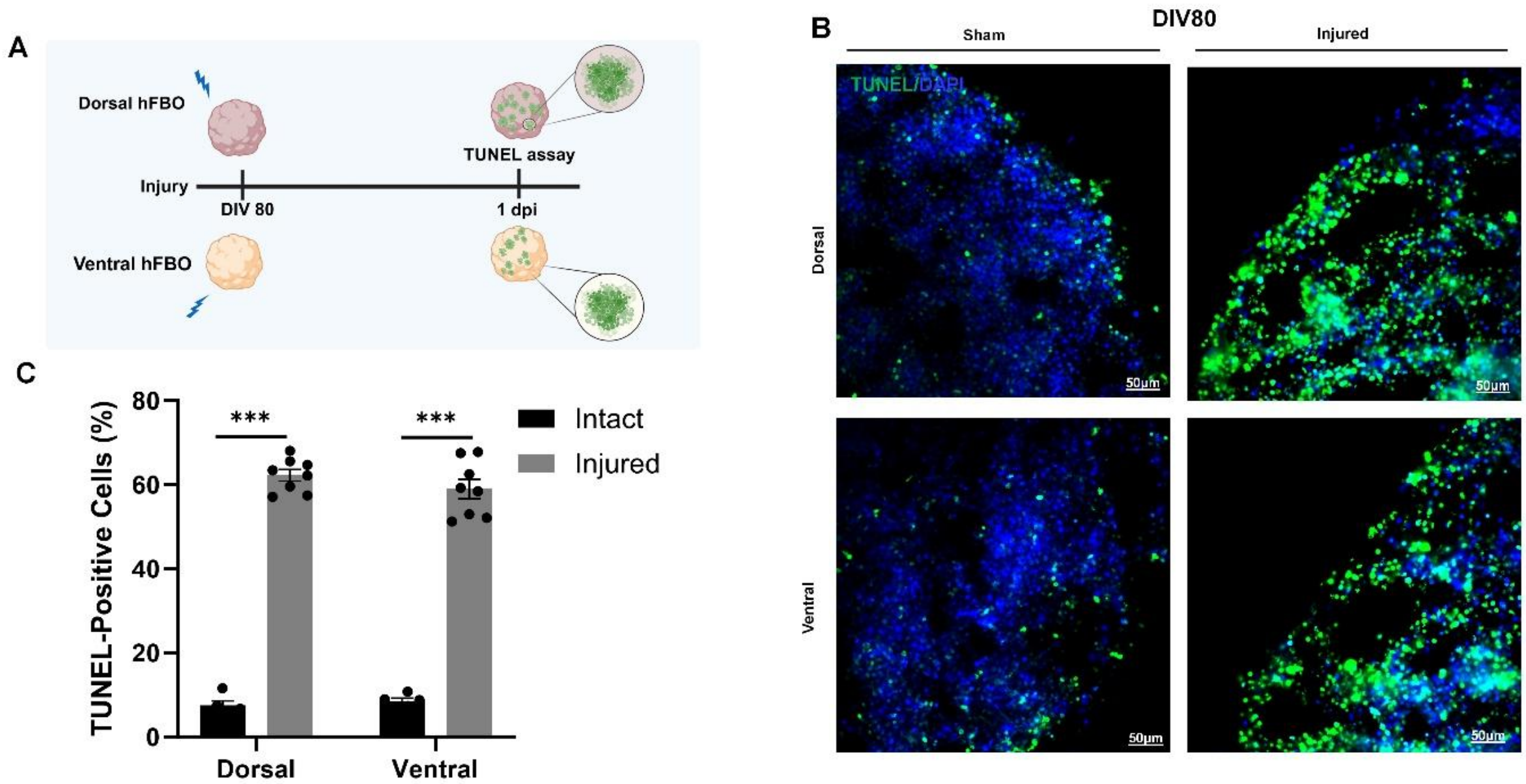

Supplementary Figure 3. Concussive injury induces comparable cell death in dorsal and ventral human forebrain organoids at 80 DIV. (A) Schematic of the experimental paradigm. Dorsal and ventral hFBOs at 80 DIV were subjected to concussive injury, and cell death was assessed by TUNEL staining at 1 day post-injury (dpi). **(B)** Representative TUNEL staining of intact and injured dorsal and ventral hFBOs. TUNEL-positive cells are shown in green and nuclei (DAPI) in blue. **(C)** Quantification of TUNEL-positive cells demonstrates a significant increase in cell death following injury in both dorsal and ventral hFBOs, with no significant difference between regional identities. TUNEL-positive cells were quantified as the percentage of TUNEL-positive cells relative to total DAPI-positive nuclei. Data are presented as mean ± SEM; n = 5 bins for intact and 8 bins for injured groups from 3 hFBOs/group derived from 3 independent batches across 1 hiPSC and were analyzed by two-way ANOVA with injury status and regional identity as factors, followed by Tukey's multiple-comparisons post hoc test. *** P < 0.001.

Supplementary table 1. Antibodies

Antibodies	Source	Identifier
Mouse anti-SOX2	Abcam	Cat# ab79351; RRID: AB_10710406
Rabbit anti-Nestin	Sigma-Aldrich	Cat# SAB4200347
Mouse anti-ZO-1	Invitrogen	Cat# 33-9100; RRID: AB_2533147
Rabbit anti-PAX6	MBL International	Cat# PD022
Mouse anti-S100β (clone 4C4.9)	Invitrogen	Cat# MA5-12969; RRID: AB_10983883
Mouse anti-βIII-tubulin (TUJ1)	BioLegend	Cat# 801202; RRID: AB_2313773
Rat anti-CTIP2	Abcam	Cat# ab18465; RRID: AB_2064130
Rabbit anti-GABA	Invitrogen	Cat# PA5-32241; RRID: AB_2549714
Rabbit anti-Parvalbumin (PV)	Invitrogen	Cat# PA1-933; RRID: AB_2173898
Rabbit anti-Somatostatin (SST)	Invitrogen	Cat# PA5-82678; RRID: AB_2789834
Rabbit anti-Ki67	Thermo Fisher Scientific	Cat# MA5-14520; RRID: AB_10979488
Mouse anti-β-catenin (clone 9F2)	Invitrogen	Cat# MA1-2001; RRID: AB_326078
Mouse anti-TLR4 (clone 25)	Santa Cruz Biotechnology	Cat# sc-293072; RRID: AB_10611320
Mouse anti-NF-κB	Santa Cruz Biotechnology	Cat# sc-8008; RRID: AB_628017
Mouse anti-Tau	EMD Millipore	Cat# 05-803; RRID: AB_310013
Goat anti-mouse IgG Alexa Fluor 488	Invitrogen	Cat# A-11001; RRID: AB_2534069
Goat anti-mouse IgG Alexa Fluor 568	Invitrogen	Cat# A-11031; RRID:
Goat anti-rabbit IgG Alexa Fluor 488	Invitrogen	Cat# A-11008; RRID: AB_143165
Goat anti-rabbit IgG Alexa Fluor 594	Invitrogen	Cat# A-11037; RRID: AB_2534095
Goat anti-rat IgG Alexa Fluor 488	Invitrogen	Cat# A-11006; RRID: AB_2534074
